# “Pumping iron”—how macrophages handle iron at the systemic, microenvironmental, and cellular levels

**DOI:** 10.1007/s00424-017-1944-8

**Published:** 2017-03-01

**Authors:** Manfred Nairz, Igor Theurl, Filip K. Swirski, Guenter Weiss

**Affiliations:** 1 Department of Internal Medicine VI, Infectious Diseases, Immunology, Rheumatology, Pneumology, Medical University of Innsbruck, Anichstr. 35, 6020 Innsbruck, Austria; 2 Center for Systems Biology, Massachusetts General Hospital and Harvard Medical School, Boston, MA USA; 3 Department of Radiology, Massachusetts General Hospital, Harvard Medical School, Boston, MA USA

**Keywords:** Macrophage, Iron, Infection, Inflammation, Erythrophagocytosis

## Abstract

Macrophages reside in virtually every organ. First arising during embryogenesis, macrophages replenish themselves in the adult through a combination of self-renewal and influx of bone marrow-derived monocytes. As large phagocytic cells, macrophages participate in innate immunity while contributing to tissue-specific homeostatic functions. Among the key metabolic tasks are senescent red blood cell recycling, free heme detoxification, and provision of iron for de novo hemoglobin synthesis. While this systemic mechanism involves the shuttling of iron between spleen, liver, and bone marrow through the concerted function of defined macrophage populations, similar circuits appear to exist within the microenvironment of other organs. The high turnover of iron is the prerequisite for continuous erythropoiesis and tissue integrity but challenges macrophages’ ability to maintain cellular iron homeostasis and immune function.

This review provides a brief overview of systemic, microenvironmental, and cellular aspects of macrophage iron handling with a focus on exciting and unresolved questions in the field.

## Systemic aspects of iron recycling

**Fig. 1:**
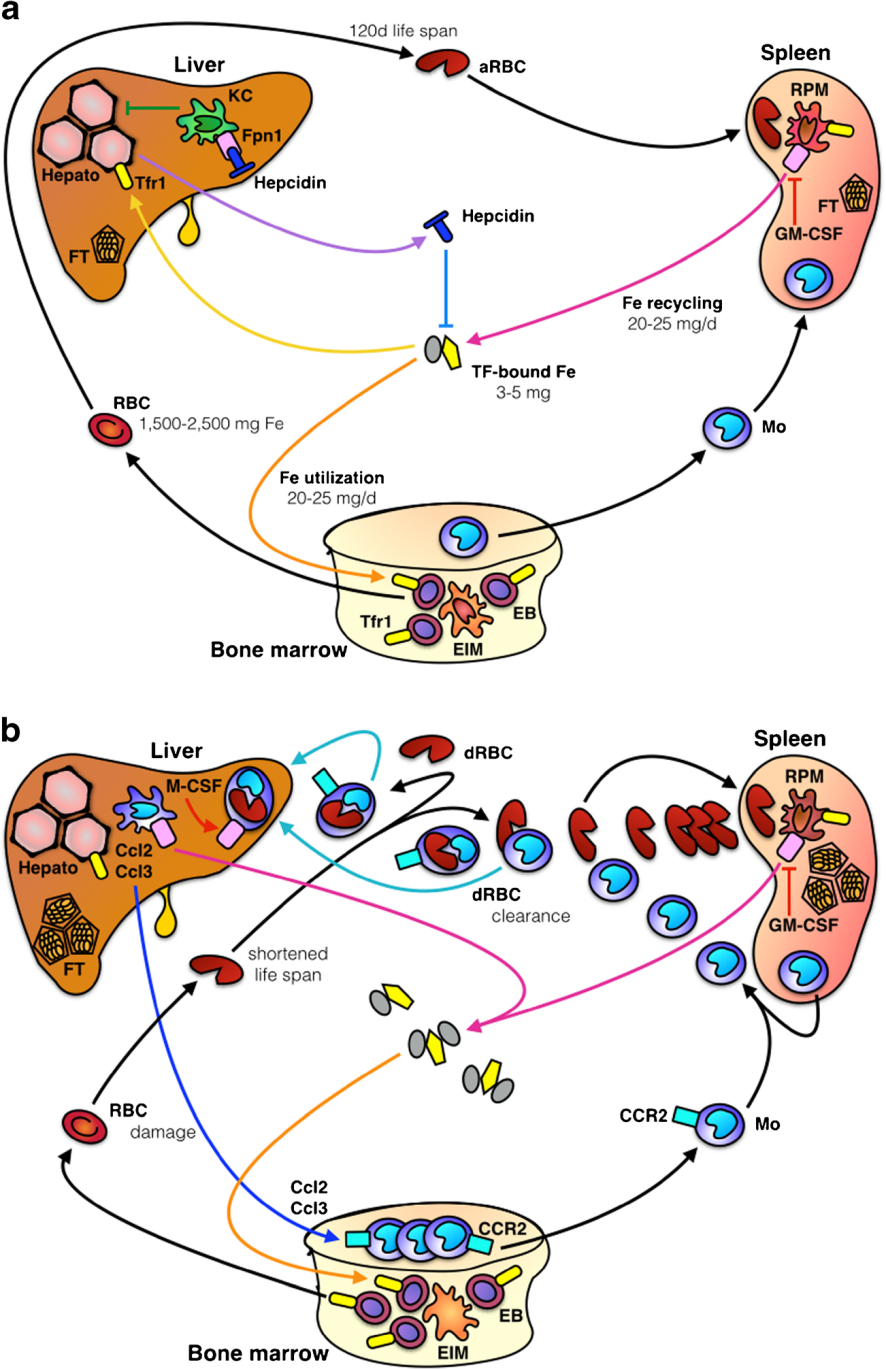
**a** In steady state, 20–25 mg/day of iron is needed to generate new red blood cells (RBC) in the bone marrow (depicted as vertebral body). The RBC of an adult human form the body’s largest iron pool (1500–2500 mg contained in hemoglobin [Hb]). After an average lifetime of 120 days, aged RBC (aRBC) are trapped in the spleen, recognized by red pulp macrophages (RPM), and eliminated. Their Hb is recycled, and ferroportin (Fpn)-1 exports iron into sinusoidal capillaries where it is loaded onto transferrin (TF). TF-bound iron is utilized by developing erythroblasts (EB) via TF receptor (Tfr)-1. Erythroid island macrophages assist in iron uptake and differentiation. Plasma iron content is sensed by hepatocytes, presumably via Tfr1 and other iron-sensitive molecules. They affect the transcriptional activation of hepcidin, the major iron hormone, in hepatocytes. Hepcidin acts as negative feedback regulator of iron influx by targeting Fpn1 resulting in degradation of the latter and thus reduction of iron transfer from the diet and macrophages to the plasma. Kupffer cells (KC) inhibit hepcidin expression by hepatocytes and also engage in erythrophagocytosis. **b** The lifespan of RBC can be reduced as a consequence of autoinflammation and subsequent hemolysis, radical formation and toxin activity or based on intrinsic structural or metabolic defects. The rapid accumulation of damaged RBC (dRBC) may overwhelm the spleen’s clearance capacity. In such a scenario, KC engulf dRBC which may result in cell death. As back-up system, monocytes are recruited from the bone marrow and possibly spleen via chemokines CCL2 and CCL3 and their receptor CCR2. These monocytes encounter a niche in the liver and differentiate into KC-like cells that express Fpn1 which is induced by several mechanisms including the growth factor macrophage colony-stimulating factor (M-CSF). Fpn1-mediated iron export sustains increased erythropoiesis in the bone marrow to compensate for losses by RBC damage

The mononuclear phagocyte system (MPS) encompasses monocytes and macrophages residing throughout the body [9, 82, 85]. A central role of this multifunctional system beyond immunity and tissue repair is to control the body’s metabolic needs for iron. The maintenance of iron homeostasis at the systemic level requires iron recycling (Fig. 1), much of which is contained in senescent red blood cells (RBC). This is a prerequisite for sufficient de novo synthesis of hemoglobin (Hb) in the bone marrow (BM), a process which consumes as much as 20–25 mg of iron per day [78, 179]. The capacity of the small intestine to absorb dietary iron, in comparison to the daily needs of iron, is rather low; in humans under steady-state conditions, 1–2 mg of iron is absorbed from the diet in the duodenum and upper jejunum. Thus, dietary iron absorption only compensates for obligatory losses of the metal, mainly through desquamation of the epidermis and the intestinal epithelium, and menstrual bleeding [168].

Intestinal iron absorption is regulated, to a large extent, at the basolateral membrane of enterocytes [59, 248]. It is at this location that ferroportin-1 (Fpn1; AKA solute carrier family 40 member 1) fulfills its function as the sole cellular efflux protein for ionic iron, shuttling apically absorbed iron to the circulation, where it is oxidized and loaded onto transferrin (TF) [173]. The presence of Fpn1 in the absorptive epithelium is under negative control of hepcidin, which is mainly secreted by hepatocytes in response to high circulating and tissue amounts of iron or upon stimulation by inflammatory mediators [171, 229]. This negative feedback loop exists because of the Janus-faced nature of iron. Iron forms the central cation of hemoglobin, other heme-containing proteins (such as myoglobin and cytochrome P450 enzymes including the mitochondrial electron transport chain), and iron-sulfur cluster-containing enzymes found in most cells [179, 180]. Free labile forms of iron and heme, however, are potentially toxic and threaten tissue integrity due to their pro-oxidative properties which are largely based on the capacity of iron to non-enzymatically boost reactive oxygen species (ROS) production via Fenton chemistry [89, 119].

Given its essential role in metabolism and cellular processes such as mitochondrial respiration, an efficient and tightly controlled pathway for maintaining iron in the body must exist and likely depends on factors controlling the recognition, uptake, and degradation of erythrocytes by macrophages [113]. In steady-state conditions, RBC recycling mainly takes place in the spleen [48, 213]. In conditions of excessive RBC damage, liver macrophages (Kupffer cells, henceforward abbreviated as KC) dominate RBC clearance (as detailed in “Liver—the iron regulatory organ” section) [231].

During massive hemolysis, circulating hemoglobin (Hb) and heme are bound by hemopexin (HPX) and haptoglobin, respectively, to avoid intravasal radical formation via the catalytic action of iron [203, 235, 244]. However, when the capacity of red pulp macrophages (the iron-recycling macrophage population in the spleen, henceforward abbreviated as RPM) and KC is overwhelmed, the cells die, presumably by a specific form of cell death called ferroptosis that is induced by iron-mediated oxidative stress and lipid peroxidation [54]. To compensate, blood-derived monocytes fill the partial niche after KC loss and differentiate into a transient macrophage population that has a unique phenotype including the high expression of the CC-chemokine receptor 2 (CCR2) [231]. Indeed, monocyte recruitment from the BM to the liver is mediated by a liver-specific, KC-derived CC-chemokine gradient of CCL2 and CCL3 that attracts CCR2^+^ CCR5^+^ monocytes to the organ. As a consequence, the BM enhances myelopoiesis and releases additional monocytes to the circulation to meet the increased demand (Fig. 1b). This chemokine-mediated monocyte recruitment is essential because if disrupted pharmacologically, damaged RBC accumulate in glomerular capillaries resulting in renal failure [231]. Comparable phenomena of KC death, although necroptotic by nature, and replacement of KCs from BM-derived monocytes have been reported in *Listeria monocytogenes* infection [21] raising the possibility that the mechanism is a general stress response that can be fine tuned according to whether the initial stressor is endogenous (e.g., RBC, heme) or exogenous (e.g., a pathogen).

In theory, additional mechanisms by which macrophages may influence systemic iron homeostasis (other than its recycling) include control of dietary iron absorption or excretion. It is generally thought that no regulated pathway of iron excretion exists in the mammalian organism. Duodenal iron absorption, however, appears to rely on macrophages, albeit indirectly via modulating hepcidin production by hepatocytes (see “Liver—the iron regulatory organ” section). To the best of our knowledge, no data exist on whether lamina propria macrophages in the small intestine affect absorptive enterocyte iron transporter expression or function, although this is possible given that macrophages produce small amounts of hepcidin in response to inflammatory stimuli [185, 227]. However, one might speculate that intestinal infection might alter absorption by shifting intestinal stem cell differentiation towards non-absorptive progeny such as Paneth or Tuft cells and by promoting recruitment of inflammatory macrophages [39, 64]. Although hypothetical, such a mechanism might be initiated by myeloid cells since lamina propria macrophages and dendritic cells (DCs) will be one of the first cell types to engage in host response to invasive pathogens [125].

### Erythrophagocytosis

The physiologic senescence of RBC is associated with mechanical and biochemical alterations of their membrane such as reduced flexibility, exposure of phosphatidylserine on their surface, and clustering of band 3 followed by binding of antibodies and complement [97, 237]. The microanatomy of splenic sinusoidal capillaries prevents the unhindered passage of these senescent RBC. Macrophages, however, recognize cell surface markers of senescence via scavenger receptors such as Tim4, SR-AI, and CD36 [114, 155, 156, 202, 251]. Damaged and aged RBC are engulfed by RPM (flow cytometrically phenotyped as F4/80^low^ CD11b^low^ Fpn1^+^) and phagocytosed. The molecular machinery of RBC recycling is relatively well described, and its function is essential to tissue integrity given the pro-oxidative capacity of free labile heme. This capacity is potentiated by the extraordinarily high number of about 1.2 × 10^9^ heme moieties contained within a single RBC and the fact that RBC are the body’s most abundant cell type comprising approximately 84% of our cells [118, 208]. It thus appears conceivable that a stand-by mechanism for heme detoxification exists: The molecular switch to turn on the protective pathways within cells is the transcriptional repressor Btb and Cnc homology 1 (Bach1). Bach1 is a basic leucine zipper (bZip) transcriptional factor which has a high affinity for heme, enabling it to act as a sensor of cellular heme levels. Rising heme levels induce Bach1 proteosomal degradation, releasing the brake on two types of down-stream pathways. First, the competitive inhibition of nuclear factor (erythroid-derived 2)-like 2 (Nrf2) binding to antioxidant-response elements (ARE) ceases for genes containing AREs [51, 145, 221], such as ferritin h chain (FTH), FPN1, heme-regulated gene-1 (HRG1), and heme oxygenase (Hmox1) [102, 190]. Second, the degradation of Bach1 induces expression of the transcription factor (an E26 transformation-specific transcription factor of the Spi subfamily) SpiC independently of Nrf2 [116, 211]. SpiC is a transcription factor that is predominately expressed by RPM and erythroid island macrophages (EIM) in the BM [88]. It is essential to the development of these cell types as *Spic* ^*−/−*^ mice have drastically reduced numbers of RPM and EIM which lead to reduced proerythroblast numbers in the steady state, supporting the idea that iron recycling is essential to sustain iron-sufficient erythropoiesis [58].

Hypoxia-inducible factors (HIFs) provide an alternative mechanism to adjust heme iron recycling by macrophages to the degree of anemia and the need for de novo Hb synthesis. Anemia inevitably results in low oxygen concentrations in the tissue microenvironment which counteract HIF degradation in the proteasome so that these transcription factors become available to trans-activate genes containing hypoxia-responsive elements (HREs). HIF-HRE interaction in response to hypoxia thus stimulates iron release from macrophages by inducing Hmox1 and Fpn1 as well as one of its associated ferroxidases, ceruloplasmin [37, 63, 200]. Although many genes involved in heme iron recycling are thus HIF-inducible, HIF-1α and HIF-2α in macrophages are dispensable for heme iron recycling under hemolytic stress [147]. This suggests that HIF isoforms fulfill redundant functions in macrophages or that they are not essential because Bach1-controlled pathways are dominant.

Bach1 is not the only cellular sensor for heme. Intracellular free heme can also induce signaling via nuclear receptors Rev-Erbα and Rev-Erbß [29, 132, 187]. Furthermore, the inflammasome activation via cryopyrin (AKA NLRP3) is sensitive to surplus intracellular heme while extracellular free heme can activate Toll-like receptor 4 (TLR4), supporting the idea that free heme is an endogenous danger signal [60, 65, 66, 131]. Given its inherent potential toxicity, the majority of free intracellular heme is promptly degraded by Hmox. Hmox1, which itself is induced via Nrf2 in response to heme or other oxidative stressors, is widely expressed in the MPS. Hmox2, by contrast, is a constitutive isoform carrying important functions in the nervous and reproductive systems [89]. Both Hmox isoenzymes cleave heme’s protoporphyrin ring which yields bilirubin, carbon monoxide, and iron in equimolar amounts. The iron released during this process is either stored within ferritin (FT), incorporated into iron-containing proteins, or shuttled to the extracellular space via Fpn1. Outside the cell, ferrous iron needs to be oxidized to its ferric form by cell membrane-anchored hephaestin or plasmatic ceruloplasmin. Thereafter, ferric iron can be bound to transferrin (TF) and the bulk of it will re-enter the BM and be utilized in RBC production. Therefore, the incorporation of iron into protoporphyrin IX to form a new heme moiety closes the circle of heme iron recycling.

## Local circuits

### Liver—the iron regulatory organ

The liver acts as central orchestrator of iron metabolism through various mechanisms. First, among many other carrier proteins, hepatocytes synthesize TF to cargo ferric iron in the circulation and keep free ionic iron at minimal levels. Second, even small fluctuations in plasma iron such as those originating from a single dose of an iron supplement are sensed by the liver and counterbalanced by an accordant change in hepcidin expression [158]. An increase in plasma iron stimulates hepcidin secretion which dampens macrophage iron egress and to a lesser extent duodenal iron absorption following its interaction with Fpn1. Conversely, KC appear to dampen hepatocytes’ hepcidin expression since KC depletion with clodronate liposomes augments hepcidin and leads to a dramatic drop in plasma iron levels according to one study [228]. However, other data suggest that KC are dispensable for the induction of hepcidin in response to iron or inflammation. Studies in *Il-6* ^*−/−*^ mice confirmed that IL-6 is an important mediator to induce hepcidin in response to TLR4 ligation but KC depletion did not alter hepcidin induction in this model [137]. While the physiological role of these observations therefore remains controversial [137, 157], it is tempting to speculate that KC may fine-tune hepcidin production or that a liver-intrinsic circuit reports on the amount of iron acquired by KC through erythrophagocytosis. A diphtheria toxin-mediated depletion of C-type lectin domain family 4 member F (Clec4F), a marker specific to KC, might help to test this hypothesis [207].

Hepatocytes are also central effectors of the acute phase reaction that is induced during extracellular hemolysis and thereby produce haptoglobin and HPX which are rapidly consumed. Haptoglobin and HPX scavenge free Hb and heme, respectively, and are ligands to CD163 and CD91 which are primarily present on macrophages including KC [100, 120]. The destruction of large amounts of RBC in the circulation further activates F4/80^+^ CD11b^low^ KC to respond with erythrophagocytosis. KCs have a limited tolerance to iron toxicity and die in the setting of massive hemolysis as discussed above [231].

Erythrophagocytosis augments Fpn1 and FT expression by F4/80^+^ CD11b^+^ liver-infiltrating monocytes that differentiate to macrophages which transiently replace dying KC. In the liver, M-CSF stimulates Fpn1 expression while GM-CSF, which is produced in the spleen, represses Fpn1 [231]. Upon differentiation, these F4/80^+^ CD11b^+^ Fpn1^+^ monocytes also express SpiC and bone morphogenetic protein 6 (Bmp6). While SpiC may program them to recycle iron efficiently, the iron-inducible factor, Bmp6, may have paracrine effects on adjacent hepatocytes by instructing them to increase hepcidin expression, thereby lowering circulating iron levels. However, the major production of Bmp6 in the liver is by yet another liver-resident cell type, sinusoidal endothelial cells [28]. Since Bmp6 stimulates both hepcidin production by hepatocytes [6] and transcriptionally regulates macrophage effector functions [123], it may broadly orchestrate the interplay between various liver-resident cell types. Intriguingly, there might be direct transfer of iron from macrophages to hepatocytes after erythrophagocytosis because iron contained in damaged RBC first accumulates in myeloid cells but is distributed to hepatocytes within 1 week. The underlying mechanism remains elusive but may employ Fpn1 as well as alternative routes of iron trafficking via FT secretion and uptake via FT receptors [34, 90, 130]. In fact, it has been shown that macrophages are the major source of plasma FT [40].

The importance of Fpn1 for liver cell functions is evident from the clinical manifestations of ferroportin disease (type 4 hemochromatosis) which is caused by various mutations in the *SLC40A1* (encoding FPN1) gene [148, 189]. Classical loss-of-function mutations of *SLC40A1* result in reduced iron transport capacity that are attributable to an amino acid exchange in the iron pore or to protein misfolding of FPN1. The mutations mainly manifest in the MPS (i.e., by iron retention in KC) because the high iron turnover of macrophages cannot be met by 50% of functional FPN1 molecules transcribed from the intact *SLC40A1* allele [58, 149]. However, non-classical Fpn1 mutations *SLC40A1* are gain-of-function mutations that cause hepcidin resistance of the FPN1 protein. In this case, hepcidin cannot bind to FPN1 and does not cause its internalization and lysosomal degradation. Consequently, a phenotype of functional hepcidin deficiency that is similar to classical type I hemochromatosis with hepatocellular iron overload occurs [2, 188, 246]. In summary, hepatocytes, KC, and endothelial cells cooperate to maintain the body’s iron homeostasis and we are beginning to decipher the signals that coordinate the function of these and other liver-resident cell types.

### Spleen—iron recycler in steady state

Similar to the liver, several cell populations co-exist in the spleen. RPM ingest erythrocytes in steady state, eliminating more than 2 × 10^6^ of these cells per second in a healthy adult (as deduced from the RBC production rate assuming equilibrium). This enormous task has implications for the spleen’s immune function. For instance, CD47 is a “do not eat me” signal expressed by many cell types including RBC [178]. CD47 is recognized by signal regulatory protein alpha (SIRPα) on macrophages which then ignore the intact RBC. In contrast, CD47-deficient RBC are rapidly cleared from the circulation. Aging RBC partially lose surface expression of CD47 [109]. Given that CD47 specifically and substantially inhibits FcγR-mediated phagocytosis of IgG-carrying RBC, the CD47 reduction with senescence is thought to enable the elimination of aged RBC. RPM are not the only ones that recognize CD47, though. The absence of CD47 on RBC is also sensed by splenic CD4^+^ DCs. Lacking the inhibitory input of CD47-SIRPα interaction, they are licensed to augment MHC-II, CD86, and CCR7 and start their migration to the T cell zone to stimulate T cells [253]. However, it remains unclear whether or not erythrophagocytosis by RPM and CD4^+^ DC activation affect each other.

In response to increased demand for erythrophagocytosis, the spleen also produces abundant amount of GM-CSF, which inhibits Fpn1 expression in macrophages [231]. It has thus been proposed that the emergency mechanism of iron recycling is less efficient under stress conditions in the spleen. This fundamental difference between spleen and liver may be based on the fact that iron alters the binding affinity of antibodies: Both ferrous iron and free heme interact with IgG to enlarge the antibody repertoire. Specifically, heme-IgG complexes have a broader ability to recognize bacterial antigens and kill intact bacteria. In addition, Fe-IgG complexes protect from *E. coli* sepsis suggesting another possible link between erythrophagocytosis and adaptive immunity [52, 53]. Another aspect underlying reduced erythrophagocytosis in the spleen under stress conditions including infections can be attributed to the fact that iron loading of macrophages dampens their innate anti-microbial effector function directed against invading pathogens [212]. While the function of RPM in continuous erythrophagocytosis is clear, we are lacking a comprehensive understanding of how iron recycling in the splenic red pulp and the organ’s immune functions affect each other.

### Muscle—venue for a closed circuit for iron recycling following myolysis?

A mechanism comparable to hemolysis-induced RBC recycling in the liver has recently been described in the skeletal muscle in the context of rhabdomyolysis. Rhabdomyolysis, the death of the skeletal muscle, poses a fundamental risk for the body’s integrity because myoglobin, the oxygen transfer protein, is a heme-containing protein that can contribute to iron-mediated radical damage. Additionally, myoglobin’s molecular weight of 17 kDa allows it to penetrate the glomerular filtration apparatus of the kidney which operates at a cutoff of 30–50 kDa so that in rhabdomyolysis large quantities of myoglobin end up in the renal tubuli where they can cause acute kidney injury (crush kidney). Therefore, the skeletal muscle is equipped with a machinery that protects from free myoglobin/heme-mediated damage. This includes HPX formation and myeloid cell recruitment. Macrophages infiltrating the injured skeletal muscle induce the expression of Hmox1, CD163, and FT to contain and detoxify heme [41]. It has further been suggested that the induction of Hmox1 and Fpn1 by the infiltrating monocytes allows for local heme iron recycling so that the regenerating myoblasts have enough iron for de novo myoglobin synthesis. It will be interesting to see whether myeloid cells also affect iron metabolism of skeletal muscle during more physiological conditions such as growth and whether there are perturbations in myopathies other than rhabdomyolysis.

### Heart—an organ exquisitely sensitive to iron toxicity

The presence of a local mechanism of iron recycling by monocytes in skeletal muscle raises the question whether similar circuits exist throughout the body. Given that myocardial ischemia induces substantial recruitment of monocytes from blood, BM, and spleen to the site of damage and its vicinity, these cells may also encounter myoglobin molecules. However, the adult myocardium lacks regenerative potential suggesting that iron handling by monocytes infiltrating the injured heart muscle may be different from that of monocytes infiltrating damaged skeletal muscle [92]. Alternatively, it is possible that cardiac fibroblasts use iron recycled by monocytes for proliferation and collagen synthesis [80].

Resident cardiac macrophages are one of the major non-parenchymal cells of the heart [223]. They have well-established roles in the pathogenesis of ischemic myocardial injury as well as in tissue repair thereafter [93, 96]. In addition, their roles in the metabolic activity of the adjacent myocardium and in chronic cardiac diseases such as congestive heart failure are increasingly appreciated [199], and macrophages may either tailor the delivery of iron to the myocardium or accumulate and detoxify heme which may mainly arise from tissue injury [161, 214].

The susceptibility of the heart to pathological iron loading is well reflected in Friedreich’s ataxia, an autosomal recessive disease with a complex neurological phenotype that inevitably affects the heart; congestive heart failure is the most frequent cause of death in these patients. Frataxin localizes to mitochondria where it fulfills functions in the assembly of iron-sulfur clusters acting as the mitochondrial iron chaperone [24].

Given the extraordinary energy generation by mitochondria in cardiomyocytes, it is likely that the cardiac phenotype of Friedreich’s ataxia is primarily a parenchymal one that occurs as a consequence of mitochondrial heme accumulation due to defective iron utilization [98]. However, it is interesting to note that the number of CD68^+^ macrophages is increased in the myocardium of Friedreich ataxia patients and that these cells contain higher amounts of FT. It has been proposed that Fpn1-mediated iron export from cardiac macrophages may be impaired in Friedreich’s disease [115].

Since cardiomyocytes are exquisitely sensitive to iron overload and iron overload disorders commonly affect the heart, a better understanding of iron handling by resident or recruited myeloid cells in the cardiac microenvironment may open new therapeutic options. However, cardiac macrophages are already an established diagnostic target of contrast material in magnetic resonance imaging (MRI). Specifically, nanoscale iron-containing compounds (IONs, for iron oxide nanoparticles) have been designed to being selectively taken up by macrophages [183]. After exposure to magnetic fields, these compounds cause a signal alteration which is affected by processes such as ischemia or inflammation secondary to macrophage activation.

### Blood vessels—arterial macrophage iron overload as hallmark of atherosclerosis

Iron accumulation in arterial macrophages has long been suggested to contribute to the pathogenesis of atherosclerosis [220]. A comprehensive pathophysiological understanding of this observation is still pending. However, it is well documented that arterial macrophages, typically identified by CD68 histochemistry on histologic sections within atherosclerotic lesions, contain larger amounts of FT than those outside plaques [129, 256]. Accordingly, high levels of plasma FT are an independent risk factor for the severity of carotid artery disease [110, 111], and high circulating iron levels have been linked to impaired endothelial function and intima media thickening, two early predictors of atherosclerosis [76]. Published evidence suggests that microhemorrhages occur in the vasa vasora of the arterial wall and that macrophages remove RBC debris by phagocytosis, which increases their iron levels without overt activation [22, 87, 201]. In addition, atherosclerosis is a lipid-driven chronic inflammatory disorder closely linked to the metabolic syndrome, and higher plasma hepcidin levels are associated with vascular damage as documented in a small but well-conducted ultrasonographic pilot study [238]. That said, hepcidin levels do not predict an increased risk for myocardial infarction or stroke in a population-based study [182].

The specific role of macrophage iron efflux in the progression of atherosclerosis is controversial. While the presence of the hypofunctional *flatiron* mutation of *Slc40a1* in apolipoprotein E-deficient (*Apoe* ^*−/−*^) mice did not affect atherosclerosis [107], systemic administration of the hepcidin-antagonist LDN-193189 (LDN), which inhibits Bmp-induced hepcidin transcription [10, 27, 230], blocked the differentiation of macrophages into foam cells. This latter effect was based on enhanced expression of ATB-binding cassette transporters ABCA1 and ABCG1, two major cholesterol efflux proteins, and on increased cholesterol transfer to apolipoprotein A1 (ApoA1) [198]. Importantly, LDN treatment also reduced atherosclerotic lesion size, suggesting that modulating systemic iron metabolism affects atherosclerosis progression. A more selective approach that only targets arterial wall macrophages may be warranted as the metabolic syndrome can be associated not only with functional iron deficiency (predicted to be ameliorated by hepcidin antagonism) but also with iron overload (predicted to be aggravated by systemic hepcidin inhibition).

The dysregulation of iron metabolism in arterial macrophages is not limited to the hepcidin-Fpn1 axis. In humans, expression of the ferroxidases hephaestin and ceruloplasmin was reduced in atherosclerosis [105]. Accordingly, there is in vitro evidence that iron-induced ROS contribute to LDL oxidation and that oxidized LDL particles induce FTH and hepcidin transcription in macrophages which may reduce Fpn1 levels and thus retain cellular iron [144].

In conclusion, evidence is accumulating for a cross-talk between the regulators of iron and cholesterol metabolism both systemically and locally in the microenvironment of the atherosclerotic plaque. However, due to a lack of mechanistic insight, we are not able yet to therapeutically target one metabolite or metabolic pathway in the attempt to beneficially affect the other.

### Brain—cerebral iron overload is implicated in neurodegeneration

In the central nervous system (CNS), macrophages appear as microglia and are in close proximity to neurons, astrocytes, and oligodendrocytes. The microglia depend on the transcription factors PU.1 (encoded by *SPI1*) and “Spalt-like”-1 (Sall1) [25] and are important for maintaining CNS homeostasis in physiological conditions and for restoring it after injury [84]. This paradigm has also been addressed in the context of iron metabolism. For instance, after experimental intracranial hemorrhage (ICH), microglia clear RBC but may also sustain inflammation and cause secondary damage. It is thus conceivable that the molecular machinery for RBC degradation and heme iron recycling including Hmox1 and Nramp1 [213] is essential for microglial function [19]. Indeed, long-term induction of Hmox1 promotes the resolution of ICH [260]. However, this may not be a feasible therapeutic approach to be readily translated into clinical practice because various cerebral cell types differ in their cytoprotective enzyme repertoire and thus in their sensitivity to iron-induced damage [255]. Cell type-specific overexpression of Hmox1 in astrocytes, although neuroprotective in some in vitro models [240] and in ICH in young mice, is deleterious in other settings [133, 215, 216] and results in spontaneous cerebral iron deposition and consecutive movement and psychiatric abnormalities with aging [36, 218]. When the iron chelator deferoxamine (DFO), however, is administered early after experimental ICH, microglial activation is blunted and reduced local concentrations of tumor necrosis factor (TNF) and interleukin (IL)-1ß are associated with protection from neuronal death. Therefore, early DFO treatment in experimental ICH translates into improved neurologic outcome [222].

Local iron accumulation in the brain (particularly in neurons and astrocytes) is linked to several neurodegenerative disorders, collectively referred to as neurodegeneration with brain iron accumulation (NBIA) [127]. Although iron-mediated generation of ROS and injury to mitochondria are likely mechanisms [196, 204], the interplay between microglia, neurons, and other cell types in cerebral iron homeostasis is incompletely characterized. A paradigmatic yet rare NBIA condition is the neuroferritinopathy that is caused by a mutation in the coding region of the *FTL* gene [44, 45]. Although FTH carries the ferroxidase activity that is required to oxidize ferrous iron and to contain it within the FT shell, FTL facilitates the mineralization of ferric iron thus supporting FTH activity. The composition of FT is cell type-specific, and central neuronal FT are L chain-rich [236]. In addition, mutated FTL proteins may have a dominant negative effect on FT’s stability and resistance to oxidative damage [12, 140, 160]. It would appear that neuronal iron efflux is insufficient to remove surplus and potentially harmful iron from the cytoplasm. Whether or not a transfer of iron between neurons and microglial cells exists is unclear. However, it has been proposed that the siderophore-binding peptide lipocalin-2 (NGAL, Lcn2) is secreted by activated microglia and other cell types including neurons to transfer iron across cellular membranes. Notably, Lcn2 also affects a variety of brain functions from cognition to emotional stress [104]. Interestingly, the hyperferritinemia-cataract syndrome that is caused by a mutation in a specific non-coding region of the *FTL* gene, the iron-responsive element (IRE), is not associated with iron accumulation in the brain but with the formation of ferritin crystals in the ocular lenses, resulting in cataracts in early adulthood [23, 83, 195].

Several common neurodegenerative disorders such as Alzheimer’s and Parkinson’s diseases are also characterized by iron deposition, but they are not considered as classical NBIA. Whether or not the latter is causatively involved in the pathophysiology of the disease or a consequence of neurodegeneration is currently not known, as the influx of iron-rich inflammatory cells and their subsequent death may contribute to neuronal iron overload [233]. In addition, an intrinsic defect of neurons in cellular iron handling may exist. For instance, in neuronal cells exposed to amyloid beta peptide fragments, iron chelation with DFO or deferiprone (DFP) reduces ROS generation via the NADPH oxidase [181]. Apart from neurons, activated microglia can accumulate iron in Alzheimer’s disease, e.g., in the hippocampus as detected my ultra-high-resolution MR [258]. A similar observation has been made in the setting of the *HFE H63D* polymorphism, which is otherwise associated with type I hereditary hemochromatosis and has been linked to neurodegeneration. Mice with the homologous mutation in *Hfe* have increased FTL chain expression in microglia, suggesting iron overload [169]. This phenotype is discrepant from the iron-poor one of other Hfe-deficient macrophage populations, but the underlying mechanism remains unknown.

Similar to cardiac imaging, IONs are selectively taken up by microglia as a function of its activation state. Conceivably, microglial activation due to ischemia or inflammation results in MR signal alterations that can be used for diagnostics. However, microglia are also exquisitely sensitive to the potential toxicity of IONs because they are rapidly degraded within lysosomes and their iron catalyzes ROS generation [184]. Although hepcidin production has been observed in the brain, co-culture models suggest that microglia enhance hepcidin expression by neurons via IL-6 in a paracrine fashion [191]. These findings thus contrast to what has been shown for co-cultured primary Kupffer cells and hepatocytes inasmuch as culture supernatants from primary Kupffer cells blunted hepcidin production [228].

The published studies provide growing insight into how microglia affect brain function, local iron homeostasis, and iron-mediated neurodegeneration. The human brain is probably the most complex system known, and we need to better understand how its different cell types interact both under physiological conditions and challenges such as cognition or memory, how iron affects the function of the various cerebral cell types, and how we can potentially interfere to prevent iron from contributing to CNS disease.

### Retina—the location in which iron aggravates light damage

From an evolutionary standpoint, the retina is part of the CNS. It is thus not surprising that it handles iron similarly to the cerebrum. Since photoreceptors are sensitive to iron toxicity, local iron regulatory circuits including the hepcidin-Fpn1 axis operate in the retina [261]. Müller glial cells and endothelial cells of the retina both express Fpn1, but only the latter cell type is sensitive to hepcidin regulation. This suggests that endothelial Fpn1 is the gatekeeper for iron transport into the retina [232]. In addition, experimental light damage to the retina results in microglial activation, its migration to the outer retina, and in induction of FT H and L chains. These effects along with oxidative stress to photoreceptors can be prevented by the oral iron chelator DFP [217]. Therefore, the retina is another organ that is sensitive to iron toxicity but has evidence of endogenous mechanisms to maintain local iron homeostasis in the microenvironment.

### Bone—osteoclasts are iron-dependent

Osteoclasts are bone-resident cells of the MPS whose primary function is to absorb bone tissue in order to maintain, repair, and remodel the skeleton. Osteoclast generation requires the fusion of macrophages, the presence of the cytokines RANKL and M-CSF, and iron. Osteoclast precursors take up iron via Tfr1 and utilize six-transmembrane epithelial antigen of prostate (Steap4) as ferrireductase [262]. In addition, the transcription factor peroxisome proliferator-activated receptor-gamma coactivator 1 beta (PGC-1ß) is essential for osteoclast differentiation [101]. Apparently, both Tfr1-mediated iron uptake and PGC-1ß activation ensure mitochondrial respiration in osteoclasts as prerequisite for energy generation and proton secretion. While PGC-1ß transactivates genes of the mitochondrial respiratory chain, iron acquired via Tfr1 is incorporated into heme moieties and iron-sulfur clusters in mitochondria ensuring activity of their enzyme repertoire. Conceivably, iron overload is often associated with skeletal disease. For instance, surplus iron in thalassemic patients may promote osteoclast differentiation and function and thus contribute to bone resorption [101].

Arthropathy of the second and third metacarpophalangeal joints is a typical manifestation of type I hemochromatosis [94]. Elevated vascular cell adhesion molecule 1 (VCAM1) levels are an accurate biomarker to diagnose this condition, but whether the adhesion molecule is also involved in its pathogenesis remains unclear [170]. Of interest, circulating VCAM1 is shed by the metalloproteinase ADAM metallopeptidase domain 17 (ADAM17, AKA TNF alpha-converting enzyme (TACE)) which is activated by inflammatory stimuli [81]. In addition, membrane VCAM1 is expressed on CD169^+^ macrophages in the spleen and provides a molecular anchor to retain hematopoietic stem cells in the murine spleen, although it is unclear whether this is also relevant to arthropathy [61]. Taken together, despite the essential role of iron for osteoclast development and the clear association of skeletal disease and iron overload, we have little information about underlying mechanisms.

### Bone marrow—are erythroblasts “nursed” with iron?

Embedded in cavities and the spongiosa of bones lies the BM as organ of hematopoiesis. Erythroid island macrophages (EIM) are a population of BM-resident macrophages that support erythropoiesis as “nurse cells” [17, 192]. The smallest erythropoietic unit thus consists of a central EIM surrounded by several erythroblasts (and stromal cells). This microarchitecture is maintained by adhesive interactions because EIM depend on a set of adhesion molecules. For instance, EIM express the αV integrin which binds ICAM4 on erythroid cells, and ICAM4 KO mice have reduced numbers of erythropoietic islands [126]. Similarly, macrophage VCAM1 interacts with the integrin-α4ß1 on erythroblasts. It is well characterized that erythroblasts expel their nuclei during differentiation and that these are taken up by EIM after phosphatidylserine exposure via Tim4 and MER proto-oncogene, tyrosine kinase (MerTK) [234, 254].

The transcription factor SpiC and its down-stream effector Hmox1 are both essential for EIM differentiation, since ablation of either gene reduces EIM number [73, 88]. Less clear however remains the role of EIM and Hmox1 in local iron management in the BM and mechanisms by which EIM may assist erythroblasts in iron acquisition. One possibility is that erythroblasts acquire ionic iron via their proximity to Fpn1 on EIM. However, since Fpn1 exports only divalent iron and erythroblasts largely rely on Tfr1 (AKA CD71) to satisfy their iron demand, one can speculate that either abundant amounts of ceruloplasmin and TF (to oxidize divalent iron) are present in erythroid islands or that direct cell-to-cell transfer of iron from EIM to erythroblasts exists. However, the deletion of Fpn1 by Cre recombinase expressed under the *lysozyme M* promoter results in iron accumulation in macrophages, including BM macrophages, and mild anemia. This suggests that EIM utilize Fpn1 to export iron and thus function as iron-rich nurse cells [259]. Such a mechanism may be particularly relevant in contexts when erythropoiesis is hyperactive yet inefficient and when local hemolysis occurs in the BM (i.e., in thalassemia syndromes). Another hypothesis is that EIM act as “iron buffers” that continuously releases adequate amounts of iron even when plasma iron levels fluctuate. Yet, another idea is that despite their other essential functions EIM contribute little to the iron acquisition by erythroblasts and that the latter import any TF-bound iron the blood stream delivers. Data in support of this concept has been generated using the CD169-DTR model: The depletion of EIM (and other CD169^+^ macrophages) had only little impact on the restoration of erythropoiesis after BM transplantation. Concretely, a moderate decrease of iron supply to erythroblasts as deduced from the reduced Hb content in reticulocytes was observed [38]. In conclusion, much needs to be learned about local mechanisms and their regulation of iron shuttling from EIM to erythroblasts at various stages of their differentiation.

## The complexity of macrophage iron handling at the cellular level

**Fig. 2:**
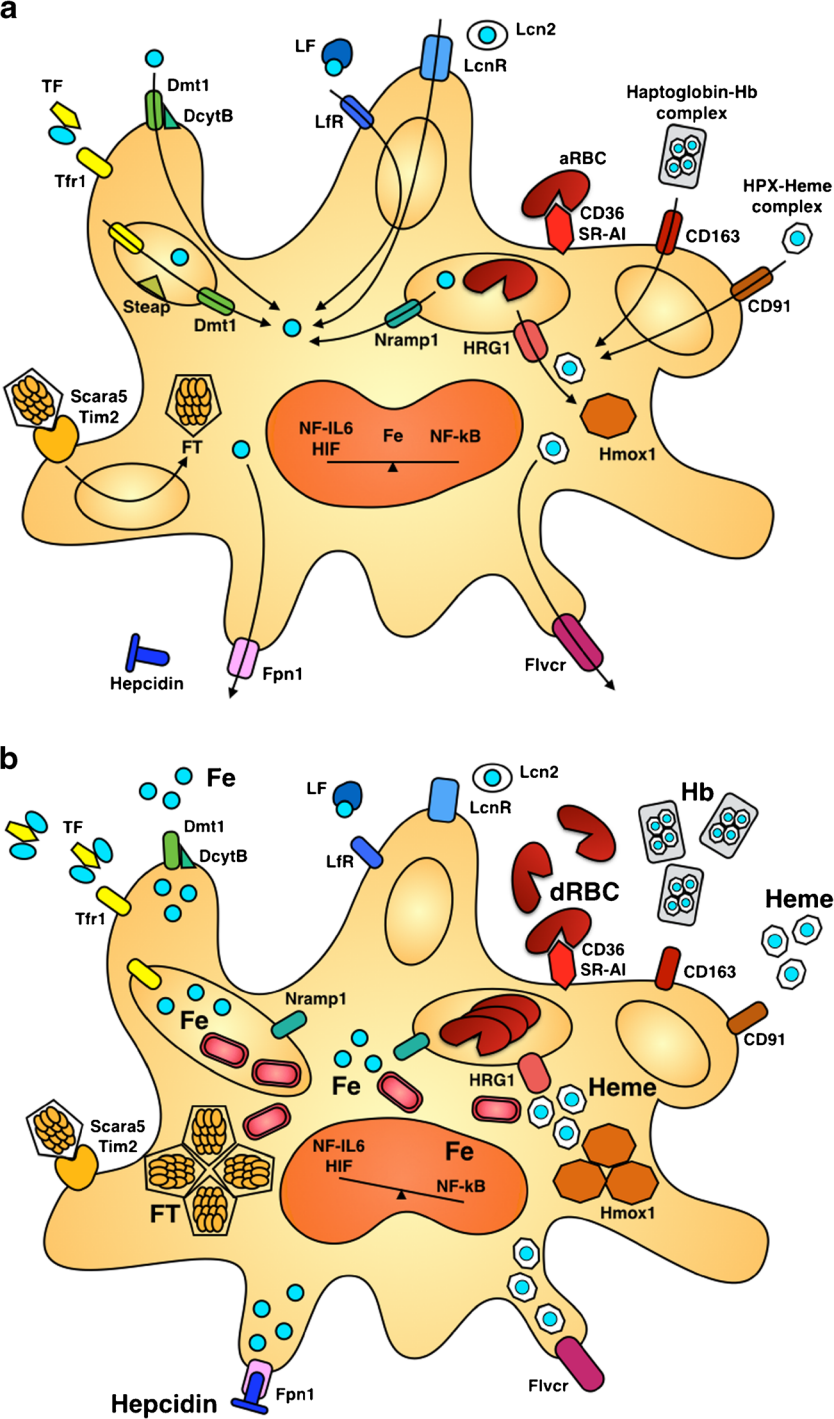
**a** In homeostatic conditions, iron uptake and release pathways are coordinated ensuring efficient iron recycling from aged red blood cells (aRBC) and iron delivery to sites of erythropoiesis. Apart from erythrophagocytosis, iron uptake mechanisms include (*left to right*) ferritin (FT) receptors such as scavenger receptor class A member (Scara)-5 and T-cell immunoglobulin and mucin domain-containing molecule (Tim)-2, transferrin (TF; depicted as monomer for simplicity) and its receptor Tfr1, the ferric reductase duodenal cytochrome B (DcytB) and divalent metal transporter (Dmt)-1, lactoferrin (LF) and its receptor LfR, lipocalin (Lcn)-2 and its receptor LcnR, haptoglobin-hemoglobin (Hb) complexes and their receptor CD163, and hemopexin (HPX)-heme complexes and their receptor CD91. Endosomal iron transporters Dmt1, natural resistance-associated macrophage protein-1 (Nramp1), and heme-regulated gene-1 (HRG1) shift ferrous iron or heme to the cytoplasm, from where the former can be exported via ferroportin (Fpn)-1 and the latter metabolized by heme oxygenase (Hmox)-1 or exported via feline leukemia virus subgroup C receptor (Flvcr). Macrophage iron homeostasis ensures proper function including the activity of pivotal transcription factors nuclear factor (NF)-IL6, NF-κB, and hypoxia-inducible factor (HIF)-1α, all of which are iron regulated. **b** Macrophage iron overload may result from an increase in circulating or local hepcidin concentrations, an elevated TF saturation, free iron excess, or hemolysis with subsequent accumulation of damaged RBC (dRBC), free Hb, or free heme. If severe, all these mechanisms will overwhelm the macrophage’s capacity to contain, store, and detoxify iron resulting in an increase of free iron, heme, and/or FT in cells. On the one hand, surplus intracellular iron may serve as nutrient for intraphagosomal or cytoplasmatic pathogens (e.g. for Gram-negative rods in the Tfr1-endosome and the cytoplasm). On the other hand, iron overload differentially affects distinct innate immune pathways. Surplus iron blocks the binding of transcription factors NF-IL6 and HIF-1α to their respective target promoter sequences. In parallel, iron facilitates the generation of ROS and thus NF-κB activation. Therefore, iron overload may cause a dysbalance in the transcriptional response to macrophage activation

Macrophages express a range of factors mediating iron uptake (summarized in the Table 1) which essentially face only two pathways for iron efflux, Fpn1 for ionic iron and feline leukemia virus subtype C receptor (Flvcr) for heme iron (Fig. 2), although alternatives routes may exist [49, 136]. One such mechanism may involve Lcn2 and one of its receptors, LcnR (AKA SLC22A17). Lcn2 binds distinct types of so-called siderophores, low molecular mass compounds with remarkably high affinity and specificity for ferric iron. These are best characterized in bacteria, but eukaryotes including fungi and mammals produce siderophores, too [16, 42, 67, 86, 106]. While some of these compounds such as citrate are ubiquitous substrates, other mammalian siderophores such as catechols are probably synthesized from commensal metabolites or dietary precursors [11, 50, 252]. Much needs to be learned about the biology of mammalian siderophores whose important functions may extend beyond mitochondrial iron homeostasis, erythropoiesis, and host defense [47, 134, 135].

**Table 1:** Selected proteins involved in macrophage iron handling

Protein	Designation(s)	Gene name	Function
TFR1	Transferrin receptor-1; CD71	*TFRC*	Uptake of TF-bound iron; essential for erythroblasts and lymphocytes [128, 141]; genetic defect associated with common immunodeficiency (IMD-46) [103]
HFE	HFE; HLA-H	*HFE*	Associates with TFR1; the *C282Y HFE* mutation causes hemochromatosis (type 1) characterized by macrophage iron depletion [18]; affects outcome of infections [13]
DMT1	Divalent metal transporter-1; Solute Carrier Family 11 member A2	*SLC11A2*	Uptake of ferrous iron through the cell surface membrane and from TFR1 endosomes; genetic defect associated with iron deficiency anemia and hepatocellular iron accumulation [68, 69, 99]
DCYTB	Duodenal cytochrome b; Cytochrome b reductase 1	*CYBRD1*	Reduction of ferric iron to its ferrous form prior to uptake via DMT1 [152, 159]; induced in iron deficiency [264]
STEAPs	Six-transmembrane epithelial antigen of prostate	*e.g. STEAP4*	Reduction of ferric iron prior to uptake via TFR1, e.g. STEAP4 in osteoclast precursors [95, 124, 177, 262]
LCNR	Lipocalin-2 receptor; 24P3R	*SLC22A17*	Bi-directional iron transport across the cell membrane requiring LCN2 and a catecholate-type siderophore [49, 210]
LCN2	Lipocalin-2; Neutrophil gelatinase associated lipocalin	*LCN2*	Binds iron-laden siderophores of different classes [11, 70]; acts as chemotaxin [206]; associated with cancer metastasis
NRAMP1	Natural resistance-associated macrophage protein-1	*SLC11A1*	Iron (and other divalent metal ion) export out of the phagolysosome for iron withholding from pathogens [14, 20, 163]; also affects odds of developing autoimmune diseases
IRP1	Iron regulatory protein-1; Aconitase 1	*ACO1*	Interaction with IREs, stabilize TFR1 and DMT1 mRNAs; blocks translation of FPN1 and FT mRNAs; its iron-sulfur cluster disassembles upon cellular iron starvation [242]
IRP2	Iron regulatory protein-2	*IREB2*	Similar to IRP1; becomes deactivated via the ubiquitin proteasome pathway when surplus iron is sensed in the cytosol [77, 243]
FTH	Ferritin heavy chain	*FTH*	Iron storage; sole carrier of the ferroxidase activity of cytosolic FT [197]; anti-apoptotic [186, 236]
FTL	Ferritin light chain	*FTL*	Iron storage; genetic defect causes neuroferritinopathy (coding region) or hyperferritinemia cataract syndrome (non-coding IRE) [3, 15, 26]
FPN1	Ferroportin-1	*SLC40A1*	Ionic iron exporter [1, 56, 150, 151, 263]; receptor for hepcidin [173]
Hepcidin	Hepcidin antimicrobial peptide	*HAMP*	Binds to FPN1 to label it for internalization and degradation; induced by IL-6, IL-22 and Bmp6 [6, 7, 172]; genetic defect results in juvenile hemochromatosis (type 2B) [174, 194]
HEPH	Hephaestin	*HEPH*	Oxidizes ferrous iron for loading onto TF [4, 33]
HRG1	Heme-regulated gene-1	*HRG1*	Shifts heme from the lysosome to the cytosol [35, 250]
FLVCR	Feline leukemia virus subgroup C receptor	*FLVCR1*	Exports heme across the cell membrane; genetic deletion results in RPM iron overload [108]

As discussed above, scavenger receptors for the uptake of senescent or damaged RBC and for the clearance of complexed and free forms of Hb and heme are expressed at high levels in liver and spleen yet may be induced at other locations in response to inflammatory stimuli. These scavenger receptors are coupled to a down-stream machinery of heme detoxification. Its two major functional components are HRG1 which shifts heme from the endosome/lysosome to the cytoplasm and Hmox1 which then metabolizes heme [117]. In addition to the phagocyte-specific scavenger receptors, Tfr1 and divalent metal transporter 1 (Dmt1), two ubiquitous iron import proteins, are also expressed by macrophages.

### A broad spectrum of iron uptake pathways

Macrophages express a range of factors mediating iron uptake, although the relative importance of these factors to different macrophage populations has not been systematically studied. It is well established that RPM and KC express the complete machinery for RBC clearance and heme iron recycling [72, 88, 231]. At either of these locations (spleen and liver), CD163 and CD91, two members of the scavenger receptor family, are expressed at high levels. Whereas CD163 binds both free and haptoglobin-bound Hb, CD91 accepts heme-HPX complexes. However, many other molecules participate in the overall process. Among these, heme-regulated gene 1 (HRG1) shifts heme from the endosome/lysosome to the cytoplasm, which is then metabolized by Hmox1 [117]. At the cell surface membrane, macrophages possess the heme exporter Flvcr and its genetic deletion results in RPM iron overload [108]. Tfr1, which is also known as CD71, is widely expressed by isolated macrophages, and it may form a major route of iron uptake in the absence of RBC (an indirect iron source), though it also appears to be important in adaptive immunity [103, 138]. Dmt1 shifts iron from the extracellular space to the cytoplasm and, in its alternately spliced form, from the Tfr1 endosome to the cytoplasm [69, 99, 139]. At either location, Dmt1 only accepts ferrous iron along with other divalent cations, such as copper or manganese, and co-localizes with an iron reductase [152, 177].

### The control of cytosolic iron levels

FT is the central mechanism of iron storage in macrophages and other cell types (25, 179, (236). However, LysM-Cre FTH mice have no apparent phenotype suggesting that iron storage and trafficking are well controlled in myeloid cells by other mechanisms [46, 239]. One such mechanism to maintain cytosolic iron levels are the iron regulatory proteins (IRPs) 1 and 2 [5, 179, 195]. IRPs sense free labile iron and control cellular iron homeostasis at the post-transcriptional level: Due to their ability to interact with iron-responsive elements (IREs) that are present in the non-coding mRNA sequences of Tfr1, Dmt1, Fpn1, FTH, and FTL chains, fluctuations in intracellular iron are promptly counterbalanced [179, 195]. Interestingly, the functions of IRPs1 and 2 are redundant in macrophages and only targeted deletion of both isoforms via the LysM-Cre system results in increased FT levels and intracellular iron retention [193]. The deletion of both IRPs in macrophages does not affect RBC recycling but impairs their nutritional immune functions against intracellular bacteria, such as *Salmonella* Typhimurium [166]. This pathogen preferentially infects murine macrophages and has a proliferative advantage in the absence of both IRPs resulting in increased bacterial burden and reduced survival in a systemic infection model. Therefore, macrophage IRPs are required to regulate their effector functions upon infection with an iron-dependent pathogen.

### Interplay of macrophage iron metabolism and immune functions

Macrophage iron metabolism and immune functions are interconnected [57, 79, 165, 247]. Clinical data as well as many studies in infection models show that macrophage iron overload, often resulting from hemolysis or iron supplementation, interferes with their antimicrobial activity (Fig. 2b). Several mechanisms explain this negative effect that surplus iron has on macrophage effector functions and the outcome of infectious diseases. First, a range of intracellular microbes can resist killing by macrophages by gaining access to macrophage iron pools [30, 245]. Second, iron alters gene transcription, translation, and mitochondrial respiration in macrophages [165, 175]. Specifically, macrophage functions driven by IFN-γ are impaired when macrophages are exposed to surplus iron [138, 162, 176] largely because iron directly inhibits the binding activities of the transcription factors nuclear factor interleukin-6 (NF-IL6) and hypoxia-inducible factor (HIF)-1α [55, 153] [176]. Because of its inhibitory effects on NF-IL6 and HIF-1α, iron excess blocks *Nos2* transcription and the high output formation of NO which worsens bacterial killing of iron-laden macrophages [91, 164]. In addition, IFN-γ in conjunction with TLR4 ligation results in NF-κB-dependent activation of HIF-1α and increased transcription of TfR1 thus aggravating macrophage iron overload [224]. Moreover, hypoxia impairs NO production by Nos2 and thus blunts the antimicrobial activity of macrophages towards *Leishmania major* [142]. Another layer of complexity is added by the fact that TLR4 signaling stabilizes HIF-1α activation via induction of FTH and hypoxia stimulates arginase-1 expression in myeloid cells thus undermining *Leishmania* killing [142, 209]. Mechanistically, these two modes of HIF activation are distinct. While TLR4 signaling results in depletion of iron as co-substrate which prolyl hydroxylases (PHDs) require to tag HIF for degradation, hypoxia, per definition, impairs PHD activity due to a lack of their substrate oxygen. Nevertheless, both hypoxia (secondary to anemia or impaired microcirculation) and the presence of TLR ligands may be present at sites of infection in close spatial or temporal association. In addition, monocytes may be exposed to fluctuating levels of both stimuli (hypoxia and inflammation) as they travel to sites of injury and infiltrate the tissue to differentiate and become sessile. Therefore, due to its sensitivity to both oxygen and iron, the transcription factor HIF-1α appears to hold a central position in the interplay of macrophage iron homeostasis and immunity.

In contrast to its inhibitory effect on NO, iron stimulates the non-enzymatic generation of ROS which may promote their antimicrobial activity but contribute to tissue damage and the pathogenesis of many inflammatory disorders. In this context, iron present in heme can stimulate the production of ROS and of pro-inflammatory cytokines such as TNF, IL-1ß, and IL-6 [241]. In contrast, arginase-1, which depletes the Nos2 substrate arginine by converting it to ornithine [62, 205], is reduced by heme. These effects may contribute to the inflammatory characteristics of sickle cell disease and can be ameliorated by the heme scavenger HPX. Accordingly, free heme, which is predicted to accumulate when Hmox1 is inhibited, activates NF-κB to induce *TNF* and *Nos2* transcription resulting in improved control of *Salmonella* infection [154]. Intriguingly, the accumulation of heme during the course of sepsis-induced hemolysis also impairs phagocyte functions due to interference with actin cytoskeleton rearrangements resulting in poor outcome [146].

A phagolysosomal transporter with homology to Dmt1 (Nramp2) is Nramp1 [8, 20, 71, 75]. Its cation transport function is directly linked to the antimicrobial activity of macrophages and DCs [74, 163, 219]. In addition, the functional form of Nramp1 in myeloid cells shifts the balance in T helper cell activity towards Th1, which may increase resistance to infections while increasing the susceptibility to autoimmune diseases [167, 226]. However, the directionality of iron transport is still under some debate [32, 71, 225, 249]: Most studies have shown that Nramp1 shifts iron and other divalent cations from the phagolysosome to the cytosol. This pathway is important for iron recycling after erythrophagocytosis [213] and serves as a mechanism of nutrient withdrawal that is particularly efficient in host defense against *Mycobacterium*, *Salmonella*, and *Leishmania* species [31]. However, some data suggest that Nramp1 in fact pumps iron into the phagolysosome where it may boost ROS generation [121, 122].

As discussed earlier, nanoparticles are increasingly used in clinical routine for imaging studies. Although intended to be otherwise inert, it has recently been uncovered in tumor models that nanoparticles may alter the biology of both tumor cells and tumor-associated macrophages, albeit with favorable results. Two elegant studies have shown that the application of nanoparticles to tumor-bearing mice is sufficient to induce clinical remission. One of these papers implicated a direct tumoricidal effect of iron delivered by ultrasmall polyethylene glycol particles and attributed tumor regression to the induction of ferroptosis in malignant cells [112]. The other study suggested that IONs stimulate TNF expression while inhibiting arginase-1 which results in improved killing of tumor cells [257]. These novel and exciting data support the general idea that the modulation of macrophage iron homeostasis is a promising way to control their immune functions and alter myeloid-driven disease processes in infection, auto-immunity, atherosclerosis, and possibly also cancer [143, 167]

## Conclusion

The MPS comprises a heterogeneous population of tissue-specific leukocytes. It is a cornerstone of innate immunity, regulates the adaptive immune system, and is indispensable for tissue development, homeostasis, and repair. One of macrophages’ central metabolic functions is to eliminate senescent and damaged RBCs and to recycle their heme-bound iron to maintain systemic and cellular iron homeostasis. The role of macrophages in iron handling preserves iron homeostasis and tissue integrity. Under physiologic conditions, macrophages recycle the iron for erythropoiesis, while under hemolytic stress, macrophages detoxify heme to prevent iron toxicity towards parenchymal cells and tissues.

Only recently, we have begun to appreciate the specific functions of various macrophage populations in the body and to study their potential roles iron metabolism. New technologies are entering the field including systems biology approaches, in situ LASER capture, single cell RNA sequencing, cell type-specific genomic editing with CRISPR/Cas9, and cell type-specific vectors to silence genes of interest [43]. Such methods will be important to critically interrogate results obtained by more drastic and unspecific approaches (such as global macrophage depletion or MAC sorting) and to understand the spatiotemporal orchestration of iron metabolism at the single cell level and in the context of the tissue microenvironment characterized by cell-to-cell contact and paracrine signals. Most of all, we face the challenge to translate findings obtained in small animal models to human subjects to improve patient care.

## Abbreviations

ABCA1, ABCG1: ATP binding cassette transporter A1 or G1, respectively
ADAM17: ADAM metallopeptidase domain 17
ApoA1, ApoE: Apolipoprotein A1 or E, respectively
ARE: Antioxidant response elements
Bach1: Btb And and Cnc Homology homology 1
BM: Bone marrow
Bmp6: Bone morphogenetic protein 6
CCL: CC-chemokine ligand
CCR: CC-chemokine receptor
CD: Cluster of differentiation
Clec4F: C-type Type lectin domain family 4 member F
Cas9: CRISPR- associated protein 9
CNS: Central nervous system
CRISPR: Clustered regulatory interspaced short palindromic repeats
DC: Dendritic cell
DcytB: Duodenal cytochrome B
DFO: Deferoxamine
Dmt1: Divalent metal transporter 1
DFP: Deferiprone
EB: Erythroblast
EIM: Erythroid island macrophages
FcγR: Fragment, crystallizable, gamma receptor
Fpn1: Ferroportin 1
Flvcr: Feline leukemia virus subtype C receptor
FT: Ferritin
FTH, FTL: Ferritin heavy chain or light chain, respectively
F4/80: (Murine macrophage marker) EGF-like module-containing mucin-like hormone receptor-like 1
GM-CSF: Granulocyte-macrophage colony-stimulating factor
Hb: Hemoglobin
HFE: Type I hemochromatosis gene
HIF: Hypoxia-inducible factor
Hmox: Heme oxygenase
HPX: Hemopexin
HRE: Hypoxia- responsive element
HRG1: Heme-regulated gene 1
ICH: Intracranial hemorrhage
IFN: Interferone
Ig: Immunoglobulin
IL: Interleukin
ION: Iron oxide nanoparticle
IRE: Iron responsive element
IRP: Iron regulatory protein
KC: Kupffer cell
Lcn2: Lipocalin 2
LcnR: Lcn2 receptor
LDN: LDN-193189 a BMP inhibitor that decreases hepcidin expression
LF: Lactoferrin
LfR: Lactoferrin receptor
MAC: Magnetic activated cell (sorting)
M-CSF: Macrophage colony-stimulating factor
MerTK: MER proto-oncogene, tyrosine kinase
MHC-II: Major histocompatibiliy complex class II
MPS: Mononuclear phagocyte system
MRI: Magnetic resonance imaging
NADPH oxidase: Nicotinamide adenine dinucleotide phosphate-oxidase
NBIA: Neurodegeneration with brain iron accumulation
Nrf2: Nuclear factor (erythroid-derived 2)-like 2
NF-IL6: Nuclear factor interleukin 6
NF-κB: Nuclear factor kappa B
NLPR3: NACHT, LRR, and PYD domains-containing protein 3 AKA cryopyrin
Nramp1: Natural resistance-associated macrophage protein 1
PHD: Prolyl hydroxylase domain proteins
PGC-1ß: Peroxisome proliferator-activated receptor-gamma coactivator 1 beta
PU.1: An E26 transformation-specific transcription factor encoded by the *SPI1* gene
RBC: Red blood cell
Rev-ErbAα, Rev-ErbBα: (Nuclear receptors) NR1D1 and NR1D2, for nuclear receptor subfamily 1, group D, member 1 or member 2, respectively
RPM: Red pulp macrophage
ROS: Reactive oxygen species
Sall1: “Spalt-like”-1
Scara5: Scavenger receptor class A member 5
SIRPα: Signal regulatory protein alpha
SpiC: An E26 transformation-specific transcription factor of the Spi subfamily
SR-A1: Scavenger receptor class A type 1
Steap: Six-transmembrane epithelial antigen of prostate
TF: Transferrin
Tfr1: Transferrin receptor 1
Tim: T-cell immunoglobulin- and mucin- domain-containing molecule
TLR4: Toll-like receptor 4
VCAM1: Vascular cell adhesion molecule 1
